# Prediction of prognosis, immunogenicity and efficacy of immunotherapy based on glutamine metabolism in lung adenocarcinoma

**DOI:** 10.3389/fimmu.2022.960738

**Published:** 2022-08-11

**Authors:** Jichang Liu, Hongchang Shen, Wenchao Gu, Haotian Zheng, Yadong Wang, Guoyuan Ma, Jiajun Du

**Affiliations:** ^1^ Institute of Oncology, Shandong Provincial Hospital, Cheeloo College of Medicine, Shandong University, Jinan, China; ^2^ Department of Oncology, Shandong Provincial Hospital affiliated to Shandong First Medical University, Jinan, China; ^3^ Department of Diagnostic and Interventional Radiology, University of Tsukuba, Ibaraki, Japan; ^4^ Department of Diagnostic Radiology and Nuclear Medicine, Gunma University Graduate School of Medicine, Maebashi, Japan; ^5^ Department of Thoracic Surgery, Shandong Provincial Hospital, Cheeloo College of Medicine, Shandong University, Jinan, China

**Keywords:** lung adenocarcinoma, glutamine metabolism, prognosis, tumor microenvironment, immunotherapy, EphB2

## Abstract

**Background:**

Glutamine (Gln) metabolism has been reported to play an essential role in cancer. However, a comprehensive analysis of its role in lung adenocarcinoma is still unavailable. This study established a novel system of quantification of Gln metabolism to predict the prognosis and immunotherapy efficacy in lung cancer. Further, the Gln metabolism in tumor microenvironment (TME) was characterized and the Gln metabolism-related genes were identified for targeted therapy.

**Methods:**

We comprehensively evaluated the patterns of Gln metabolism in 513 patients diagnosed with lung adenocarcinoma (LUAD) based on 73 Gln metabolism-related genes. Based on differentially expressed genes (DEGs), a risk model was constructed using Cox regression and Lasso regression analysis. The prognostic efficacy of the model was validated using an individual LUAD cohort form Shandong Provincial Hospital, an integrated LUAD cohort from GEO and pan-cancer cohorts from TCGA databases. Five independent immunotherapy cohorts were used to validate the model performance in predicting immunotherapy efficacy. Next, a series of single-cell sequencing analyses were used to characterize Gln metabolism in TME. Finally, single-cell sequencing analysis, transcriptome sequencing, and a series of *in vitro* experiments were used to explore the role of EPHB2 in LUAD.

**Results:**

Patients with LUAD were eventually divided into low- and high-risk groups. Patients in low-risk group were characterized by low levels of Gln metabolism, survival advantage, “hot” immune phenotype and benefit from immunotherapy. Compared with other cells, tumor cells in TME exhibited the most active Gln metabolism. Among immune cells, tumor-infiltrating T cells exhibited the most active levels of Gln metabolism, especially CD8 T cell exhaustion and Treg suppression. EPHB2, a key gene in the model, was shown to promote LUAD cell proliferation, invasion and migration, and regulated the Gln metabolic pathway. Finally, we found that EPHB2 was highly expressed in macrophages, especially M2 macrophages. It may be involved in the M2 polarization of macrophages and mediate the negative regulation of M2 macrophages in NK cells.

**Conclusion:**

This study revealed that the Gln metabolism-based model played a significant role in predicting prognosis and immunotherapy efficacy in lung cancer. We further characterized the Gln metabolism of TME and investigated the Gln metabolism-related gene EPHB2 to provide a theoretical framework for anti-tumor strategy targeting Gln metabolism.

## Introduction

Lung cancer remains the leading cause of cancer-related death worldwide (1). Non-small cell lung cancer (NSCLC) accounts for 85% of lung cancers, with lung adenocarcinoma (LUAD) constituting half of all cases of NSCLC (2). Notwithstanding the advances in treatment strategies, the five-year survival rate of patients with LUAD remains limited. In recent years, immunotherapy showed significant efficacy in LUAD, while drug resistance and recurrence due to tumor heterogeneity still limit the efficacy of immunotherapy (3, 4). Therefore, it is essential to comprehensively investigate the mechanisms underlying the differential response to immunotherapy and develop tools to predict prognosis and immunotherapy efficacy.

Recent investigations revealed that oncogenic transformation induces a well-characterized metabolic phenotype in tumor cells, which in turn affects the tumor environment (TME) (5). TME is composed of distinct cell populations in a complex matrix, which is characterized by inefficiencies of oxygen and nutrition delivery due to limited or poorly differentiated vasculature. In order to meet the energy demands, rapidly proliferating cancer cells compete with immune cells for nutrients required to manifest anti-tumor effects, thus creating an immune suppressive environment. In this harsh TME, infiltrating immune cells are forced to undergo relevant metabolic adaptations, which in turn disrupt the anti-tumor effects of immune cells (6, 7). Therefore, therapeutic strategies that target tumor metabolism and thus modulate or improve immune cell metabolism to enhance inflammation are extremely promising. However, tumor cells share a large number of metabolic pathways with inflammatory immune cells, which makes metabolic blocking strategies often counterproductive (8). Therefore, targeting the appropriate metabolic pathway to block tumor metabolism and activate inflammatory immunity is essential to improve immunotherapy. Targeting Gln metabolism is one of the optimal choices available.

As the most abundant amino acid in circulation, glutamine (Gln) is rapidly consumed by cultured tumor cells (9). Gln is commonly used to maintain TCA flux in cellular aerobic glycolysis, or as a source of citrate for lipid synthesis in reductive carboxylation. Besides, glutaminolysis also promotes survival of proliferating cells by suppressing oxidative stress and maintaining the integrity of mitochondrial membrane (10). Gln is an energy source required by both tumor and immune cells. However, inflammatory antitumor immune cells do not appear to rely on or even reject Gln metabolism, which is particularly evident in macrophages (11, 12). Compared with naïve macrophages, M2 macrophages exhibit strong dependence on Gln, while pro-inflammatory M1 macrophages can be induced by suppressed Gln metabolism. Therefore, Gln metabolism represents a potential target to convert tumor-associated macrophages (TAMs) from M2 to M1 phenotype, thereby enhancing the anti-tumor inflammatory immune response (13). In addition, Gln metabolism is also involved in the differentiation of Th1 cells and the activation of effector T cells (13, 14). These findings suggest that targeting Gln metabolism can potentially remodel TME and improve immunotherapy efficacy. In fact, recent studies reported that extensive blockade of Gln metabolism significantly improves the anti-tumor effect of anti-PD-1, accompanied by enhanced cytotoxic function of effector T cells due to metabolic reprogramming (15). In LUAD, although targeting Gln metabolism in combination with immunotherapy is extremely promising, the landscape of Gln metabolism in TME is still not fully known. Therefore, we performed this study for a systematic analysis of Gln metabolism and immunotherapy in LUAD.

Our study comprehensively evaluated the expression of Gln metabolism-related genes. Based on these genes, 514 patients with LUAD from The Cancer Genome Atlas (TCGA) were clustered using a consensus clustering algorithm and eventually a scoring system was constructed for predicting overall survival (OS) and immunotherapy efficacy. An integrated Gene-Expression Omnibus (GEO) cohort including 719 patients with LUAD and 32 cohorts of pan-cancer from TCGA were used to validate the predictive performance of the risk model. Five independent immunotherapy cohorts were identified to validate the predictive performance for immunotherapy efficacy. Multiple single-cell sequencing data were used to describe the Gln metabolism landscape of various cell types in TME. Finally, using *in vitro* experiments based on second-generation sequencing and public single-cell sequencing analysis, we investigated the regulation of biological behavior and signaling pathways of LUAD cells by EPHB2, a key gene related to Gln metabolism, which is also significantly enriched and plays an essential role in M2 macrophages.

## Materials and methods

### Data source and preprocessing

Public gene expression data (fragments per kilobase million, FPKM) and full clinical annotations were respectively retrieved from TCGA (https://cancergenome.nih.gov/) and GEO (https://www.ncbi.nlm.nih.gov/geo/) databases. The FPKM values of LUAD were transformed into transcripts per kilobase million (TPM). The training cohort included 513 patients with LUAD from TCGA while 6 eligible LUAD cohorts (GSE13213, GSE37745, GSE31210, GSE3141, GSE30219, GSE50081) from GEO dataset represented the validating cohort of our study, which consisted of 719 patients with LUAD. Pan-cancer gene expression data were extracted from TCGA for further validation.

An individual cohort with 33 LUAD specimens from Shandong Provincial Hospital was set as a validating cohort. Besides, 22 tumor specimens and 11 normal specimens from Shandong Provincial Hospital were used to perform differential expression analysis and survival analysis of EPHB2.

### Consensus molecular clustering based on Gln metabolism-related genes

73 Gln metabolism-related genes were extracted from Molecular Signatures Database (MSigDB, http://www.gsea-msigdb.org/gsea/msigdb/index.jsp). These genes are listed in **Supplementary Materials**. A consensus clustering algorithm was used to classify LUAD cohorts into distinct GlnClusters and test the corresponding stability based on survival-related Gln genes. ConsensuClusterPlus package was used to perform the above steps and 1000 repetitions were conducted to guarantee the corresponding stability.

### Identification of DEGs and construction of geneClusters

Differentially expressed genes (DEGs) among 3 GlnClusters were identified using “limma” package in R with an adjusted P value< 0.001 and a |logFC|>1. Survival-related DEGs were identified *via* univariate cox regression analysis, and patients with LUAD were classified into distinct geneClusters based on selected DEGs using R package “ConsensuClusterPlus”.

### Construction and validation of a prognostic risk model

Survival-related DEGs were sequentially subjected to Lasso Cox regression analysis and multivariate Cox regression analysis. Ten genes were finally identified and involved in the construction of the prognostic risk model, including EPHB2, CAV2, RTN2, SCPEP1, UNC5D, FURIN, PITPNC1, CH25H, RGS20 and TSPAN11. The risk score was calculated following the formula:


Risk score=∑(Expi*Coefi)

Coefi and Expi denote the risk coefficient and gene expression, respectively. Based on the median risk score of training cohort, patients from training and validating cohorts were divided into low-risk and high-risk groups, respectively. Kaplan–Meier survival analysis was followed by the generation of receiver operating characteristic (ROC) curves involving low- and high-risk groups.

### Enrichment analysis and functional annotation

Gene Set Variation Analysis (GSVA) enrichment was performed to explore the heterogeneity of various biological processes using “GSVA” package. Hallmark gene sets “h.all.v7.4.symbols.gmt” extracted from MSigDB database were used for GSVA. R package “ClusterProfiler” was applied to perform functional annotation. Single sample gene set enrichment analysis (ssGSEA) was performed to calculate the score of Gln metabolism based on 73 previously extracted Gln metabolism-related genes.

### Mutation and drug susceptibility analysis

The mutation annotation format (MAF) from the TCGA database was generated using R package “maftools” and the somatic mutations of LUAD in low- and high-risk groups were plotted. The tumor mutation burden (TMB) of each patient with LUAD in the TCGA cohort was also calculated. Drug sensitivity analysis was performed with R package “pRRophetic”. A parliament plot was developed to demonstrate drug sensitivity of low- and high-risk groups using the website HIPLOT (https://hiplot.com.cn/).

### TME landscape analyses

Single sample gene set enrichment analysis (ssGSEA) was performed to calculate and compare the enrichment scores of infiltrating immune cells and immune function (16, 17). Immune score, ESTIMATE score and stromal score were calculated using the ESTIMATE algorithm (18). Data of T cell dysfunction, T cell exclusion and TIDE scores were obtained from TIDE website (http://tide.dfci.harvard.edu/). A correlation heatmap of 10 genes in the risk model and 4 panels of immune function were also downloaded from the TIDE website (19). Immunophenoscore (IPS) of patients in TCGA was obtained from The Cancer Immunome Atlas (https://tcia.at/).

### Immunotherapy datasets

Five immunotherapeutic cohorts were used to validate the prediction of immunotherapy efficacy using the risk model: melanoma treated with adoptive T cell therapy (ACT) (GSE100797) (20); melanoma treated with pembrolizumab, an anti-PD-1 antibody (GSE78220) (21); melanoma treated with anti-CTLA4 and anti-PD1 therapy (GSE91061) (22); NSCLC treated with nivolumab or pembrolizumab, an anti-PD-1 antibody (GSE126044) (23); and advanced urothelial cancer treated with atezolizumab, an anti-PD-L1 antibody (IMvigor210 cohort) (24). The response and benefit of TCGA cohort were calculated based on the TIDE website (http://tide.dfci.harvard.edu/) by integrating TIDE score, INFG, MSI score, CD274, Merck18, CD8, MDSC, CAF and TAM M2.

### Establishment and validation of a nomogram scoring system

A predictive nomogram was constructed using R package “rms”, which consisted of risk, age and stage. The total score of each patient was calculated based on each variable matched score. The calibration plot was used to assess the predictive value between the predicted 1-, 3-, and 5-year OS rates and the actual results observed. Time-dependent ROC curves were plotted to assess the prediction of 1-, 3-, and 5-year OS by the nomogram.

### Single-cell RNA-seq analysis and online website analysis

GSE111907 was used to evaluate the degree of Gln metabolism in malignant, pan-immune cells, endothelial and fibroblast cells. GSE117570, GSE131907, GSE99254 and GSE127465 were analyzed in the website scTIME Portal (http://sctime.sklehabc.com/unicellular/home) (25). The degree of Gln metabolism was calculated using ssGSEA based on 73 identified Gln metabolism-related genes.

The differential expression analysis of 10 pan-cancer genes was performed online at Gene Expression Profiling Interactive Analysis (GEPIA, http://gepia.cancer-pku.cn/).

### Transcriptome sequencing

Transcriptome sequencing was performed in EPHB2-siRNA treated PC-9 cells using the Illumina NovaSeq platform with Annoroad Gene Technology. The differentially expressed genes were identified with FC > 2 and P< 0.05.

### RNA extracting and real-time PCR

Following manufacturer’s protocol, the total RNA of LUAD specimens or cells was extracted using the AG RNAex Pro Reagent (Accurate Biotechnology (Hunan) Co., Ltd China). The cDNA was synthesized after reverse transcription using Evo M-MLVRT Master Mix kit (Accurate Biotechnology (Hunan) Co., Ltd China). The relative gene expression was detected using the SYBR Premix Ex Tap kit (Accurate Biotechnology (Hunan)Co.,Ltd China) and normalized to the expression using 18S. The primers are listed in **Supplementary Table 1**.

### Cell culture and reagents

Human PC-9, A549 and THP-1 cell lines were purchased from Procell Life Science & Technology Co., Ltd. PC-9 and THP-1 cells were maintained in RPMI 1640 (Gibco) supplemented with 10% FBS, and A549 cells were maintained in F12K (Gibco) supplemented with 10% FBS. The cell lines were cultured at 37°C in a humidified incubator containing 5% CO_2_.

### EPHB2 knockdown

PC-9 cells were plated at a density of 3*10^5^ per 60 mm dish. After 24 h culture, the medium was changed to fresh medium. The PC-9 cells were transfected with EPHB2-short interfering RNAs (siRNAs) or control-siRNA purchased from TransheepBio (Shanghai, China), accompanied by jetPRIME^®^ transfection reagent (PolyPlus transfection, Illkirch, France). The transfected cells were cultured for at least 24 h in 10% FBS RPMI 1640 medium. The sequences of the EPHB2 siRNA were as follows: 5’GGGAAAUACAAGGAAUAUU3’ (si1), 5’CGCUUUCUAGAGGACGAUA3’ (si2), 5’GGAGUUUGCCAAGGAAAUU3’ (si3) and 5’GAUGAUGAUGGAGGACAUU3’ (si4).

### Proliferation assay

Cells were seeded in 96-well plates at a density of 1500 cells per well. After at least 6 hours, the first dish was fixed with 10% cold trichloroacetic acid for at least 24 hours, and the other plates were fixed every 24 hours. After washing and drying, the plates were stained with Sulforhodamine B sodium salt (SRB) (Sigma, USA) for 20 minutes and washed with 1% (vol/vol) acetic acid. After drying, 150 µL of 10 mmol/L Tris was added and the absorbance was measured using the microplate reader (Thermo Fisher, USA) at 562 nm. The results were analyzed with GraphPad Prism 8.0.2.

### Colony formation assay

Cells were seeded in 6-well plates at a density of 500 cells per well and cultured at 37°C for two weeks. Subsequently, the plates were washed with phosphate-buffered saline (PBS) and fixed with 4% paraformaldehyde for 30 minutes. Finally, 0.1% crystal violet was used to stain the plates. The colonies were counted with ImageJ software (Wayne Rasband, National Institutes of Health, USA).

### Wound healing assay

Cells were seeded in 12-well plates and monolayers were scratched with a pipette tip until 95% confluence. The cells were subsequently photographed every 12 hours and the migrated areas were calculated using ImageJ software.

### Transwell assay

Cells (4×10^4^) were seeded in the upper chamber in RPMI 1640 without FBS. The lower chamber was filled with 600 µL of RPMI 1640 medium containing 20% FBS. After 24 hours of incubation, the cells were fixed and stained with crystal violet. The cells in the upper chamber were removed, the migrated cells were photographed and counted with ImageJ software.

### THP-1 polarization

THP-1 cells were seed into 6-well plates and treat with PMA (Sigma-Aldrich, St. Louis, MO, USA) for 48 h. Then cells were treated with IL-4 (20 ng/ml; PeproTech) for 24h to induce M2-phenotype polarization.

### Immunofluorescence (IF)

IF assay was implemented according to the methods described previously (26). The primary antibodies included EPHB2 (1:100, 2D12C6, Santa Cruz Biotechnology) and CD206 (1:100,24595, Cell Signaling Technology).

### Western blot analysis

Protein samples were dissolved in lithium dodecyl sulfate (LDS) sample buffer (Invitrogen). Equivalent amounts of total protein extract were separated on 10% SDS-PAGE gels (90 V for 30 min and 120 V for 60 min) and transferred to polyvinylidene fluoride membranes. The transfer was carried out at 100 V for 2 h using a Bio-Rad transfer apparatus. Membranes were then blocked for 1 h at room temperature in 5% BSA solution. Appropriate primary antibody was incubated overnight at 4°C. The primary antibodies were listed as followed: Akt, p-Akt (Ser473), ERK1/2 and p-ERK1/2 (Thr202/Tyr204) (Cell Signaling Technology, USA: 1:1000); GAPDH and EPHB2 (Santa Cruz, USA: 1:1000).

### Statistical analysis

The statistical analysis of this study was performed using R-4.1.2 software. For quantitative data, the statistical significance of normally distributed variables was estimated by the Student’s t-test, and non-normally distributed variables were analyzed using the Wilcoxon rank sum test. Comparisons between more than two groups were made using the Kruskal-Wallis test and one-way analysis of variance as non-parametric and parametric methods, respectively. Kaplan-Meier survival analysis was performed with the R package “Survminer”. Statistical significance was set as P< 0.05.

## Results

### Landscape of genetic variation of Gln metabolism-related genes in LUAD

**Figure 1:**
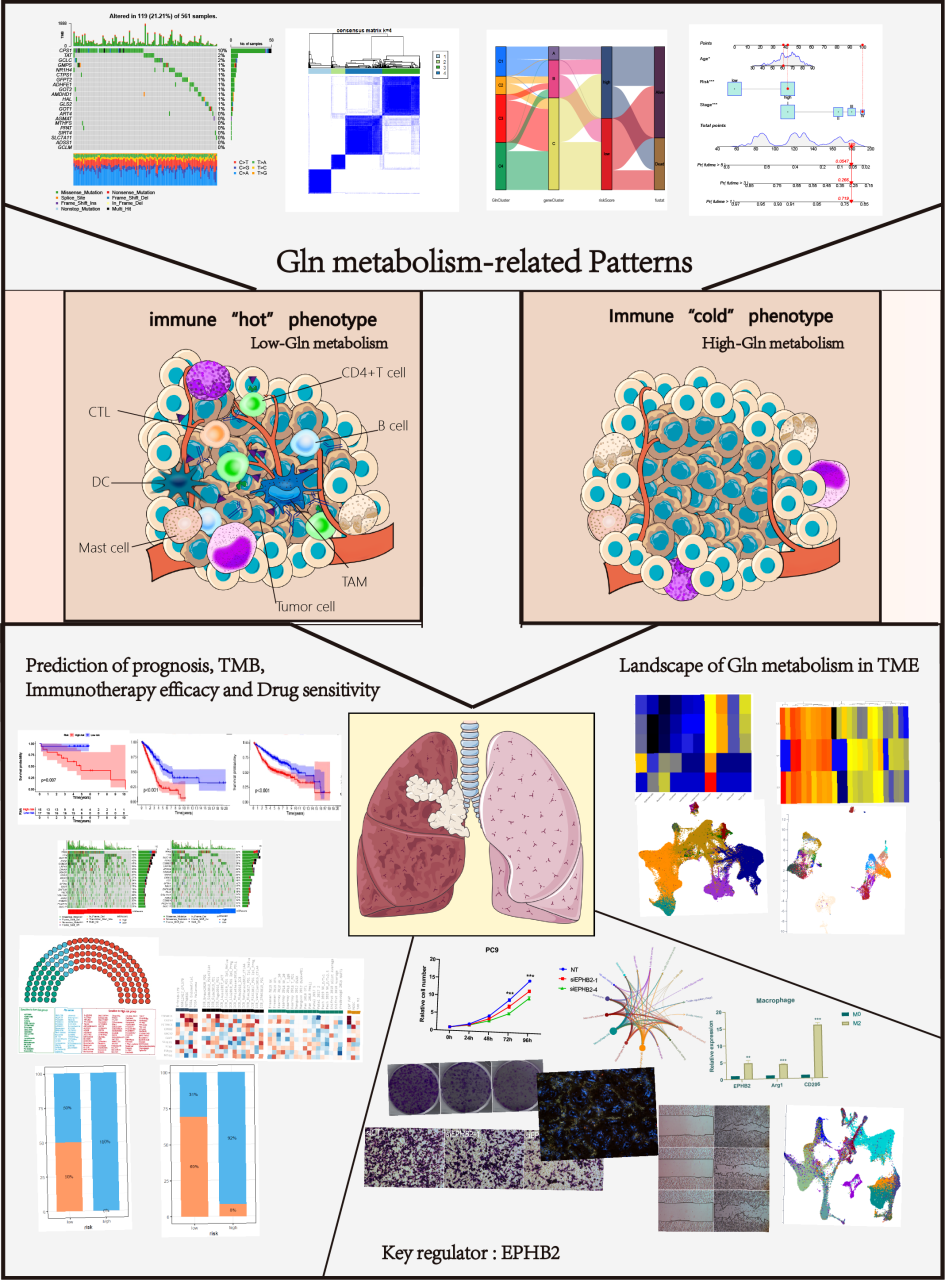
Analysis workflow of this study.

**Figure 2:**
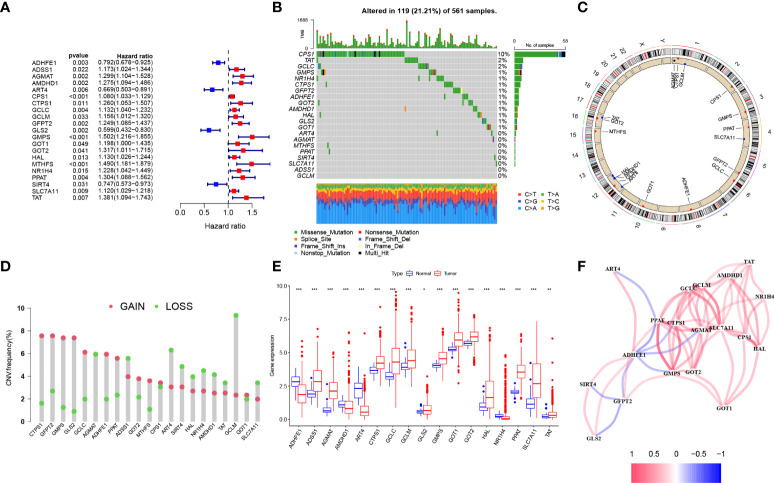
Genetic and transcriptional alterations of Gln metabolism regulators in LUAD. **(A)** Prognosis-related Gln metabolism regulators after uniCox regression analysis. **(B)** 119 of the 561 LUAD patients showed genetic alterations of prognosis-related Gln metabolism regulators. **(C)** The location of CNV alterations of prognosis-related Gln metabolism regulators on chromosomes. **(D)** CNV mutation was widespread in 21 prognosis-related Gln metabolism regulators. The column represented the alteration frequency. Deletion, green dot; Amplification, pink dot. **(E)** Differential mRNA expression of prognosis-related Gln metabolism regulators between normal and tumor samples (*P < 0.05; **P < 0.01; ***P < 0.001). **(F)** Correlation network between prognosis-related Gln metabolism regulators.

The overall design of our study is shown in the flow chart (**Figure 1**). Seventy-three Gln metabolism genes were identified from MSigDB and published articles. Based on univariate Cox regression analysis, 21 survival-related Gln metabolism genes were selected for further analyses (**Figure 2A**). A waterfall chart was plotted to show the somatic mutations of the 21 genes and the highest rate of somatic mutations in CPS1 (**Figure 2B**). The location of copy number variations (CNV) on chromosomes is shown in **Figure 2C**. The frequency of CNV amplification and deletion is displayed in **Figure 2D**. Differential expression analysis revealed that 13 genes were significantly upregulated in tumor, while 4 genes were downregulated (**Figure 2E**). The correlation network showed expression correlation between the 21 survival-related genes (**Figure 2F**).

### Construction of distinct GlnClusters

**Figure 3:**
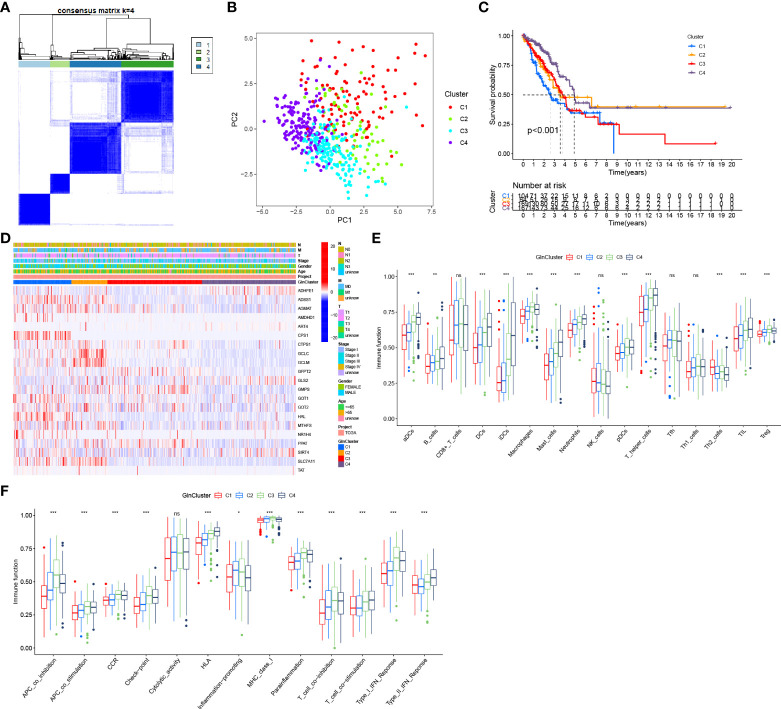
Distinct Gln metabolism-related patterns. **(A)** Consensus clustering matrix for k = 4. **(B)** Principal component analysis (PCA) for the transcriptome profiles of four clusters. **(C)** Survival analyses for four different clusters based on 513 LUAD patients from TCGA. **(D)** Heatmap of prognosis-related Gln metabolism regulators in four clusters. **(E)** The abundance of tumor infiltrating immune cells in four clusters. **(F)** The difference of immune functions between four clusters. "*” means that p < 0.05; “**” means that p < 0.01; "“***” means that p < 0.001; ns, no significance.

Based on survival-related Gln metabolism genes, 513 patients with LUAD from TCGA were stratified into 4 distinct patterns, which were defined as GlnClusters (**Figure 3A**). PCA revealed significant differences in Gln metabolism genes between the 4 clusters (**Figure 3B**). Survival analysis revealed improved prognosis of patients in cluster C4 and poor overall survival in cluster C1 (**Figure 3C**). Most of the Gln metabolism genes were significantly upregulated in clusters C1 and C2, followed by cluster C3, which implied relatively active Gln metabolism. Alternatively, cluster C4 showed reduced Gln metabolism with widespread low expression of Gln metabolism-related genes (**Figure 3D**).

We also analyzed the infiltrating immune cells and immune-related functions in different clusters. Interestingly, the abundance of most infiltrating immune cells gradually increased from clusters C1 to C4, which was inversely proportional to the Gln metabolic activity, including various DCs (aDCs, DCs, iDCs and pDCs), mast cells, neutrophils, T helper cells and TILs (**Figure 3E**). Simultaneously, APC co-stimulation, HLA, T cell co-stimulation and type II IFN response showed trends suggesting highly active antigen presentation and antitumor immunity (**Figure 3F**).

### Construction of geneClusters based on DEGs

**Figure 4:**
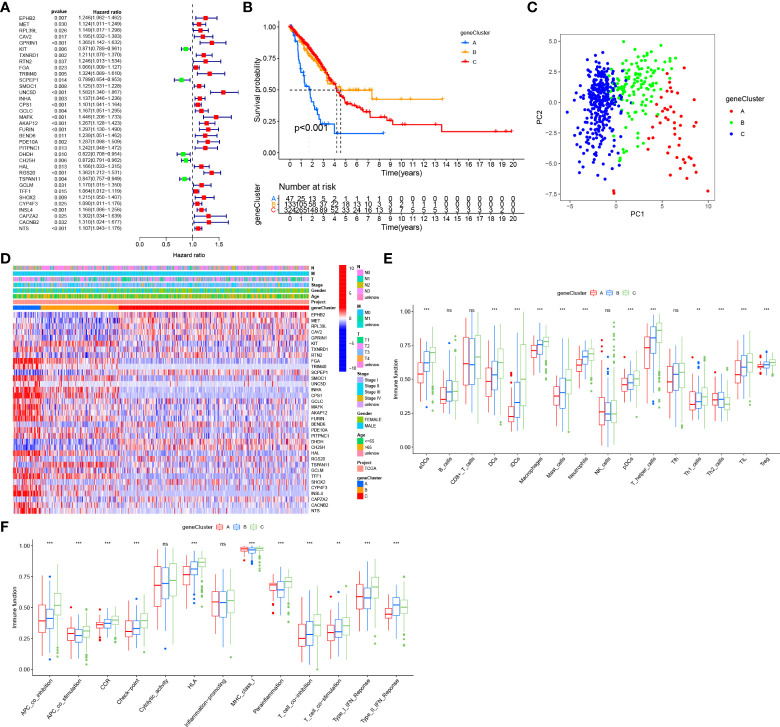
Construction of gene clusters based on DEGs. **(A)** Univariate cox regression analysis of DEGs. **(B)** Survival analyses for the three gene clusters based on the prognosis-related DEGs. **(C)** PCA for the transcriptome profiles of three gene clusters. **(D)** Expression of prognosis-related DEGs in three gene clusters. **(E)** The abundance of tumor infiltrating immune cells in three gene clusters. **(F)** The difference of immune functions between three gene clusters. “**” means that p < 0.01; "“***” means that p < 0.001; ns, no significance.

The 237 DEGs among 4 GlnClusters were screened out (P value< 0.001, |logFC|>1) and intersected with GEO validating cohort. Univariate Cox regression analysis of these DEGs was performed and 35 survival-related DEGs were identified for further analysis (**Figure 4A**). Based on the 35 DEGs, 513 patients were divided into 3 geneClusters. Compared with geneClusters B and C, the geneCluster A exhibited significant survival disadvantage (**Figure 4B**). PCA analysis revealed obvious differences in dimensions between distinct geneClusters (**Figure 4C**). A heatmap illustrated that the DEGs were significantly different between distinct geneClusters, and most DEGs were upregulated in geneCluster A (**Figure 4D**). Corresponding to the survival disadvantage, geneCluster A also exhibited a lower abundance of most infiltrating immune cells and immune functions (**Figures 4E, F**). In summary, geneCluster A can be defined as immune “cold” phenotype.

### Development and validation of a risk model

**Figure 5:**
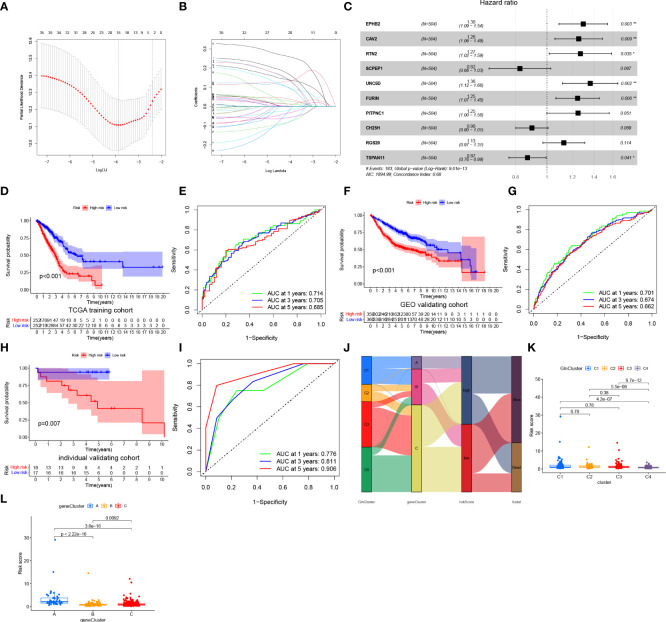
Construction and validation of a prognostic risk model. **(A, B)** Lasso regression analysis of prognosis-related DEGs. **(C)** Multivariate Cox regression analysis. **(D)** Survival analyses for low- and high-risk group in training cohort. **(E)** ROC curves of predicting prognosis in training cohort. **(F)** Survival analyses for low- and high-risk group in GEO validating cohort. **(G)** ROC curves of predicting prognosis in GEO validating cohort. **(H)** Survival analyses for low- and high-risk group in individual validating cohort. **(I)** ROC curves of predicting prognosis in individual validating cohort. **(J)** Alluvial diagram showing the relationships of survival status, Gln clusters, gene clusters and risk score. **(K)** The distribution of risk score in different clusters. **(L)** The distribution of risk score in different gene clusters. "*” means that p < 0.05; “**” means that p < 0.01.


To construct a more convenient scoring model for clinical prediction, we performed Lasso regression analysis of the identified 35 survival-related DEGs and 18 Gln metabolism-related genes remained based on the minimum partial likelihood deviance (**Figure 5A**). Subsequently, we performed multivariate Cox regression analysis of the 18 genes based on Akaike information criterion (AIC) value and 10 Gln metabolism-related genes were finally obtained, including EPHB2, CAV2, RTN2, SCPEP1, UNC5D, FURIN, PITPNC1, CH25H, RGS20 and TSPAN11 (**Figure 5B**). Based on the results of multivariate Cox regression analysis, a risk model was constructed based on the formula:

Risk score=∑(Expi*coefi)

Coefi and Expi denote the risk coefficient and gene expression, respectively.

Based on the median of risk score in training cohort, patients with LUAD from training (TCGA) and validating (integrated GEO) cohorts were divided into low- and high-risk groups, respectively. A heatmap demonstrated a high abundance of Gln metabolism-related genes in the low-risk group, suggesting the activation of Gln metabolism (**Figure 5C**). The Kaplan–Meier survival curves demonstrated a significant survival advantage of patients in the low-risk group compared with patients in the high-risk group in training (**Figure 5D**) and validating cohorts (**Figure 5F**), respectively. The area under the ROC curves (AUCs) were 0.714, 0.705 and 0.685 in TCGA training cohort and 0.701, 0.674 and 0.662 in GEO validating cohort for predicting 1-, 3-, 5-year survival times, respectively, which revealed the excellent performance of the model in predicting overall survival of patients with LUAD (**Figures 5E,G**). Besides, an individual validating cohort with 33 LUAD patients from Shandong Province Hospital was used to validate the risk model. Consistently, patients in the low-risk group revealed a significant survival advantage, compared with high-risk group (**Figure 5H**). The ROC curves indicate the excellent performance of the risk score in predicting prognosis (**Figure 5I**). **Figure 5J** illustrates the distribution of patients diagnosed with LUAD in four GlnClusters, three geneClusters and two risk groups. Compared with GlnClusters C1, C2 and C3, patients in GlnCluster C4 exhibited significantly lower risk scores (**Figure 5K**). Patients in geneCluster A exhibited the highest risk scores, while patients in geneCluster B showed the lowest risk score (**Figure 5L**).

The distribution of risk scores (**Supplementary Figures 1A, B**), survival status (**Supplementary Figures 1C, D**) and gene expression (**Supplementary Figures 1E, F**) in training and validating cohorts are presented. PCA revealed discernible dimensions between high- and low-risk groups in training and validating cohorts, respectively (**Supplementary Figures 1G, H**).

### TMB and drug susceptibility analysis

**Figure 6:**
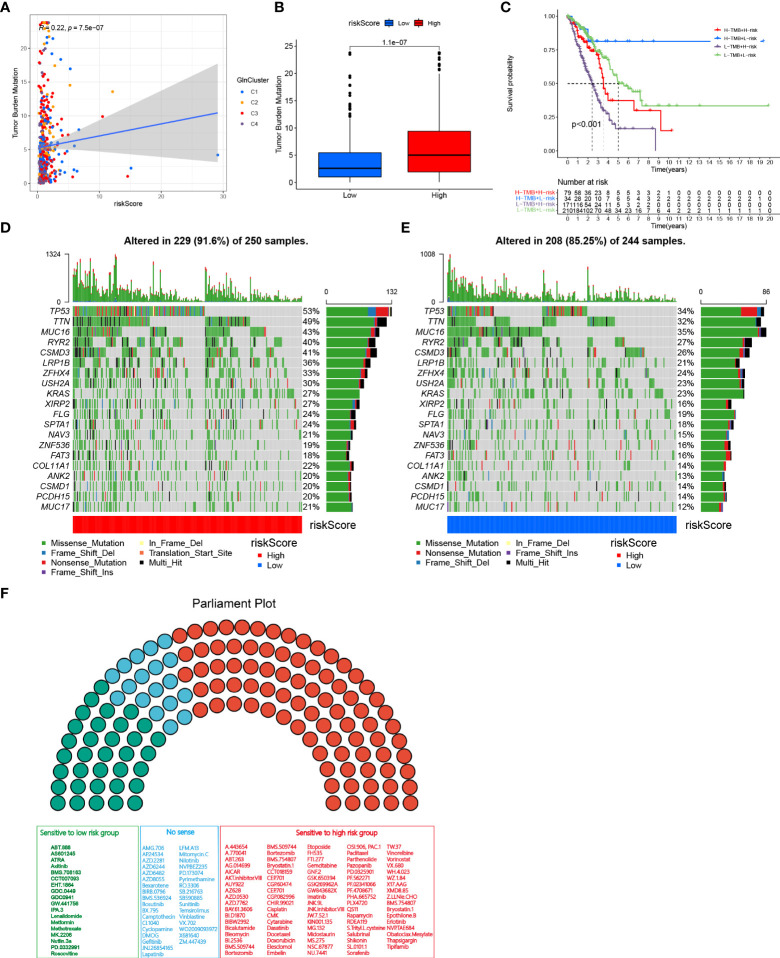
TMB and drug susceptibility analysis. **(A)** Correlation analysis between risk score and TMB. **(B)** Difference between low and high-risk group. **(C)** Kaplan–Meier curves show overall survival differences stratified by TMB and risk score (p < 0.001). Visualization of gene mutations in high-risk group **(D)** and low-risk group **(E)**. **(F)** Drug sensitivity analyses between low-and high-risk groups. Green, sensitive to patients with low risk scores; Red, sensitive to patients with high risk scores; Blue, no sense.

To investigate the correlation between risk score and TMB, Spearman correlation analysis was performed and significant positive correlation was found between risk score and TMB (R = 0.22, P< 0.001, **Figure 6A**). Patients in high-risk group had higher levels of TMB than in low-risk group (**Figure 6B**). After integrating TMB scores, patients with LUAD from TCGA were divided into four groups. Survival analysis revealed that patients with high TMB and low risk exhibited significant survival advantage, followed by the group with high TMB + low risk and low TMB + high risk, sequentially. The group with low TMB and high risk showed significant survival disadvantage (**Figure 6C**). The variation in the distribution of somatic mutations between low- and high-risk groups was investigated in the TCGA-LUAD cohort. Patients in high-risk group displayed significantly higher frequencies of somatic mutations compared with patients with low risk scores, especially in TP53 (53% vs 34%), TTN (49% vs 32%), MUC16 (43% vs 35%), RYR2 (40% vs 27%), CSMD3 (41% vs 26%) and LRP1B (36% vs 21%) (**Figures 6D, E**). We further performed drug sensitivity analysis to predict IC_50_ of 136 chemotherapy drugs (**Figure 6F**). Our results revealed that 84 drugs had lower IC_50_ values in the high-risk group, indicating sensitivity. Alternatively, patients in low-risk group were sensitive to 18 drugs. Together, these results provide a standard of reference for treatment stratification of patients with LUAD.

### Distribution of Gln metabolism and risk scores

**Figure 7:**
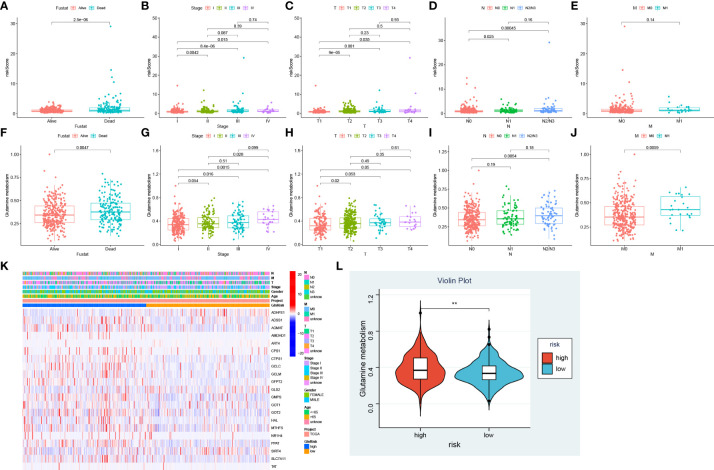
Association between Gln metabolism, risk scores and clinical characteristics. Difference of risk score between different survival status **(A)**, stages **(B)**, T stages **(C)**, N stages **(D)**, and M stages **(E)**. Level of Gln metabolism in different survival status **(F)**, stages **(G)**, T stages **(H)**, N stages **(I)**, and M stages **(J)**. **(K)** Expression of Gln metabolism regulators between low- and high-risk groups. **(L)** Difference of Gln metabolism level between low- and high-risk groups. " **” means that p < 0.01.

To determine the correlation between risk score and clinical characteristics, we evaluated the differences in risk score among different subgroups based on survival status, stage and TNM stage. Patients in alive, stage I, stage T1 and stage N0 exhibited lower risk scores compared with other groups, while there was no difference in risk score across M stages (**Figures 7A–E**). To further investigate the distribution of Gln metabolism, we performed ssGSEA to calculate the value of Gln metabolism based on 73 Gln-related genes identified. Similar to the risk score, dead patients had higher levels of Gln metabolism (**Figure 7F**). In addition, the level of Gln metabolism was significantly and positively correlated with stages T, N and M, with higher stage implying higher Gln metabolism (**Figures 7G–J**). We next analyzed the differences in Gln metabolism between low- and high-risk groups. The heatmap revealed significant upregulation of prognostic Gln metabolism-related genes in the high-risk group (**Figure 7K**). Consistently, patients with higher risk scores revealed higher levels of Gln metabolism (**Figure 7L**). In conclusion, Gln metabolism and risk scores were significantly correlated, and both were positively associated with malignant progression of LUAD.

### Evaluation of TME and prediction of immunotherapy efficacy in high- and low-risk groups

**Figure 8:**
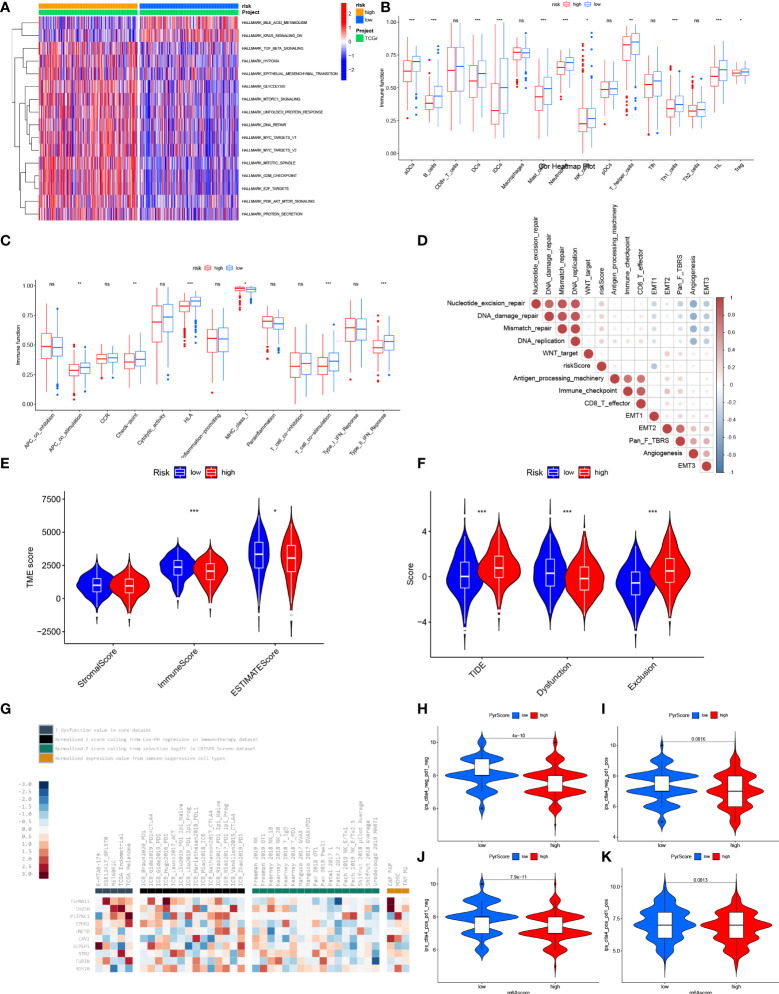
Characteristic of TME between low- and high-risk group. **(A)** GSVA enrichment analyses based on the Hallmarker gene sets showed the states of biological processes in low- and high- risk groups. **(B)** The abundance of tumor infiltrating immune cells in low- and high-risk groups. **(C)** The difference of immune functions between low- and high-risk groups. **(D)** Correlation between risk score and tumor-related functions. **(E)** Differences of ESTIMATE score, stromal score and immune score between low- and high- risk score. **(F)** Differences of T cell dysfunction, exclusion and TIDE in low- and high-risk score. **(G)** Enrichment of 10 selected genes in T cell dysfunction level, ICB response outcome, phenotypes in genetic screens and cell types promoting T cell exclusion. Difference of IPS with CTLA4- and PD-1- **(H)**, CTLA4- and PD-1+ **(I)**, CTLA4+ and PD-1 **(J)** and CTLA4+ and PD-1+ **(K)** between low- and high-risk group. "*” means that p < 0.05; “**” means that p < 0.01; "“***” means that p < 0.001; ns, no significance.

To further investigate the functional characteristics, we performed GSVA enrichment analysis of the two groups (**Figure 8A**). The results showed that bile acid metabolism was significantly upregulated in the low-risk group. Alternatively, the KRAS signaling pathway was inhibited in the low-risk group. In addition, various carcinogenic pathways were activated in the high-risk group, suggesting a possible positive correlation with Gln metabolism, such as TGF-β signaling, hypoxia, glycolysis, EMT, PI3K-AKT-MTOR signaling, DNA repair, MYC signaling and E2F targets.

To further explore the correlation between risk score and TME, we analyzed the differential abundance of immune-infiltrating cells and immune function to characterize the landscape of TME. Various immune cells involved in antigen presentation, processing and tumor killing were present at higher levels of abundance in the low-risk group, such as aDCs, B cells, DCs, iDCs, NK cells, T helper cells, Th1 cells and TIL (**Figure 8B**). Correspondingly, the low-risk group also showed active signaling of antigen recognition, processing and presentation, and antitumor effects, including APC co-stimulation, HLA, T cell co-stimulation and type II IFN response (**Figure 8C**). Besides, the low-risk group showed a higher expression of immune checkpoints, revealing possible benefit from immune checkpoint inhibitor (ICI) therapy. The risk score was also positively correlated with other carcinogenic pathways, such as nucleotide excision repair, DNA damage repair, mismatch repair and DNA replication (**Figure 8D**). A low risk score was also significantly correlated with a high immune score and ESTIMATE score, indicating increased abundance of infiltrating immune cells (**Figure 8E**). In summary, the low-risk group can be defined as a “hot” immune phenotype, associated with highly infiltrated antitumor immune cells and upregulated antitumor pathways.

**Figure 9:**
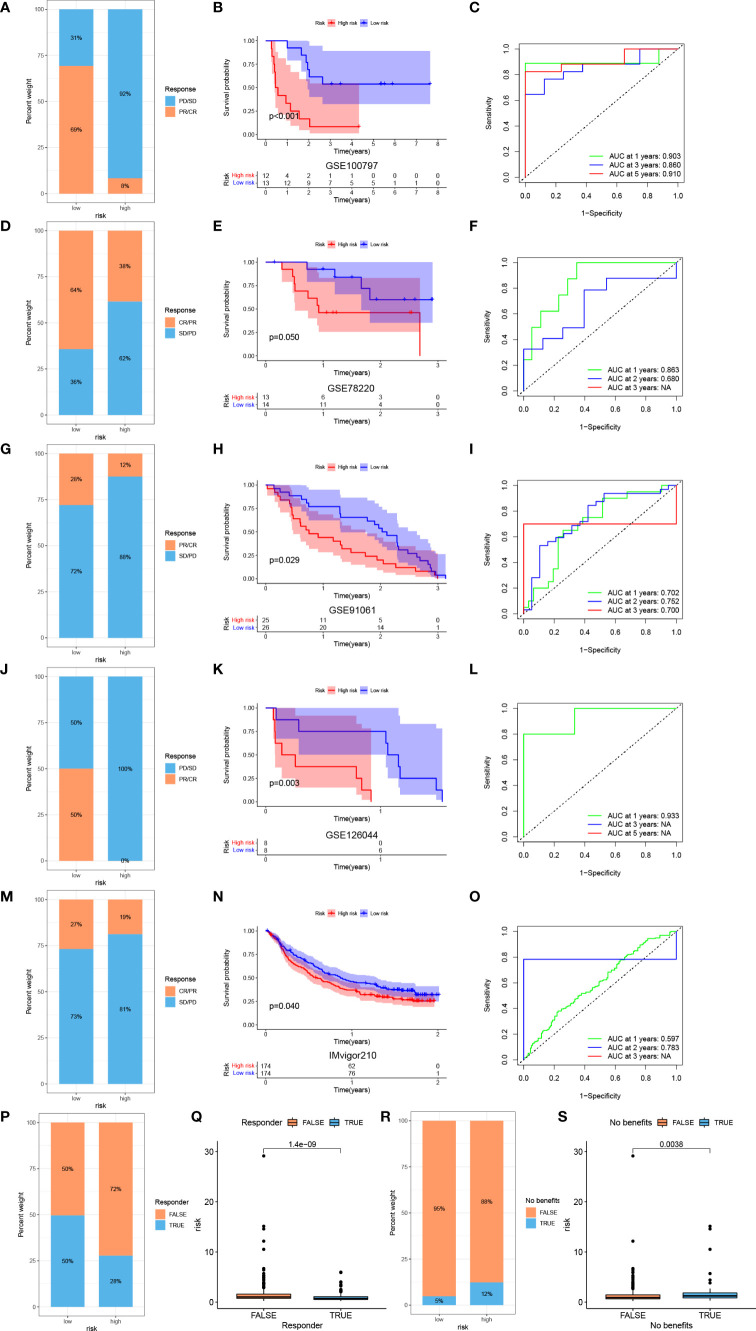
Prediction of immunotherapy efficacy by the risk model. Response to ACT **(A)**, survival analyses **(B)** and ROC curves of predicting prognosis **(C)** between low- and high-risk groups in melanoma cohort (GSE100797). Response to anti-PD-1 therapy **(D)**, survival analyses **(E)** and ROC curves of predicting prognosis **(F)** between low- and high-risk groups in melanoma cohort (GSE78220). Response to anti-CTLA4 and ant-PD1 therapy **(G)**, survival analyses **(H)** and ROC curves of predicting prognosis **(I)** between low- and high-risk groups in melanoma cohort (GSE91061). Response to anti-PD-1 therapy **(J)**, survival analyses **(K)** and ROC curves of predicting prognosis **(L)** between low- and high-risk groups in NSCLC cohort (GSE126044). Response to anti-PD-L1 therapy **(M)**, survival analyses **(N)** and ROC curves of predicting prognosis **(O)** between low- and high-risk groups in advanced urothelial cancer cohort (IMvigor210 cohort). **(P)** Difference of responder between low- and high-risk group of LUAD in TCGA. **(Q)** Difference of risk score between responder and non-responder of LUAD in TCGA. **(R)** Difference of benefits between low- and high-risk group of LUAD in TCGA. **(S)** Difference of risk score between benefit and no benefit of LUAD in TCGA.

To further investigate the correlation between risk score and efficacy of immunotherapy, we calculated the TIDE score. Patients with a low risk exhibited higher levels of T cell dysfunction and a lower level of T cell exclusion and TIDE score (**Figure 8F**). We further evaluated the association between the expression of each gene and several immunotherapy-related features, including T cell dysfunction, ICB response outcome, phenotypes in genetic screens and cell types promoting T cell exclusion (**Figure 8G**). Higher IPS was also exhibited by patients in the low-risk group compared with those in the high-risk group, which indicated that patients with a low-risk score were more sensitive to immunotherapy (**Figures 8H–K**). To fully validate the accuracy of risk score in predicting the efficacy of immunotherapy, multiple independent immunotherapy cohorts in the published literature were used to validate immunotherapy efficacy and prognosis. Melanoma treated with adoptive T cell therapy (ACT) (**Figures 9A–C**), melanoma treated with pembrolizumab, an anti-PD-1 antibody (**Figures 9D–F**), melanoma treated with anti-CTLA4 and ant-PD1 therapy (**Figures 9G–I**), NSCLC treated with nivolumab or pembrolizumab, an anti-PD-1 antibody (**Figures 9J–L**), advanced urothelial cancer treated with atezolizumab, an anti-PD-L1 antibody (**Figures 9M–O**) were used to validate the performance of risk score in predicting prognosis and efficacy of immunotherapy. Patients with a low-risk score were more sensitive to immunotherapy (**Figures 9A, D, G , J, M**). Further, patients in the low-risk group had a significant survival advantage compared with those in the high-risk group (**Figures 9B, E, H, K, N**), and the predictive performance was tested using ROC curves (**Figures 9C, F, I, L, O**). The response to anti-PD1 and anti-CTLA4 therapy was calculated using the TIDE website based on TCGA cohort (**Figures 9P–S**). Patients in the low-risk group were established as responders to immunotherapy (**Figures 9P, Q**). By contrast, patients in the high-risk group were shown to be less likely to benefit from anti-PD1 and anti-CTLA4 immunotherapy (**Figures 9R, S**).

### Prognostic validation of risk score in pan-cancer

**Figure 10:**
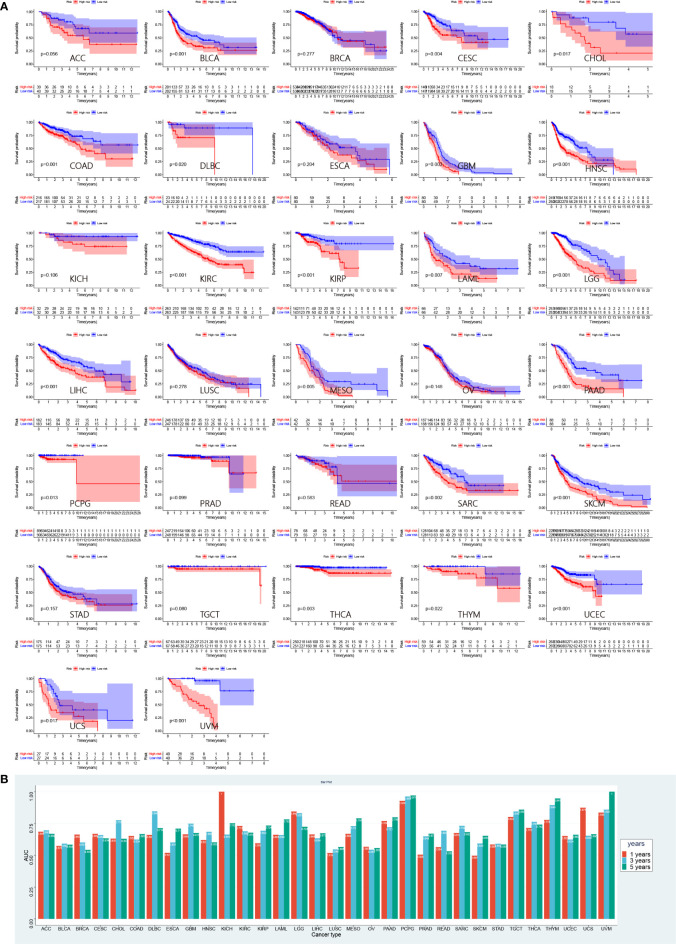
Prognostic validation of risk score in pan-cancer. **(A)** Survival analyses between low- and high-risk group in 32 pan-caner cohorts of TCGA. **(B)** Corresponding AUC values in 32 pan-cancer cohorts.

To further validate the performance of risk score in predicting prognosis of other tumors, we performed a survival analysis of patients in the high- and low-risk groups involving 32 types of tumors in TCGA other than LUAD (**Figure 10A**). Patients in the low-risk group had a significant survival advantage in 22 tumors, including bladder urothelial carcinoma (BCLA, p = 0.001), cervical squamous cell carcinoma and endocervical adenocarcinoma (CESC, p = 0.004), cholangiocarcinoma (CHOL, p = 0.017), colon adenocarcinoma (COAD, p = 0.001), lymphoid neoplasm diffuse large B-cell lymphoma (DLBC, p = 0.02), glioblastoma multiforme (GBM, p = 0.003), head and neck squamous cell carcinoma (HNSC, p< 0.001), kidney renal clear cell carcinoma (KIRC, p< 0.001), kidney renal papillary cell carcinoma (KIRP, p< 0.001), acute myeloid leukemia (AML, p = 0.007), brain lower grade glioma (LGG, p< 0.001), liver hepatocellular carcinoma (LIHC, p<0.001), mesothelioma (MESO, p = 0.005), pancreatic adenocarcinoma (PAAD, p< 0.001), pheochromocytoma (PCPG, p = 0.013), sarcoma (SARC, p = 0.002), skin cutaneous melanoma (SKCM, p< 0.001), thyroid carcinoma (THCA, p = 0.003), thymoma (THYM, p = 0.022), uterine corpus endometrial carcinoma (UCEC, p< 0.001), uterine carcinosarcoma (UCS, p = 0.017) and uveal melanoma (UVM, p< 0.001). The ROC curves were performed to evaluate the prognostic performance of pan-cancer risk scores (**Supplementary Figure 2**). The AUC values are presented in **Figure 10B**.

### Development of a nomogram to predict survival

**Figure 11:**
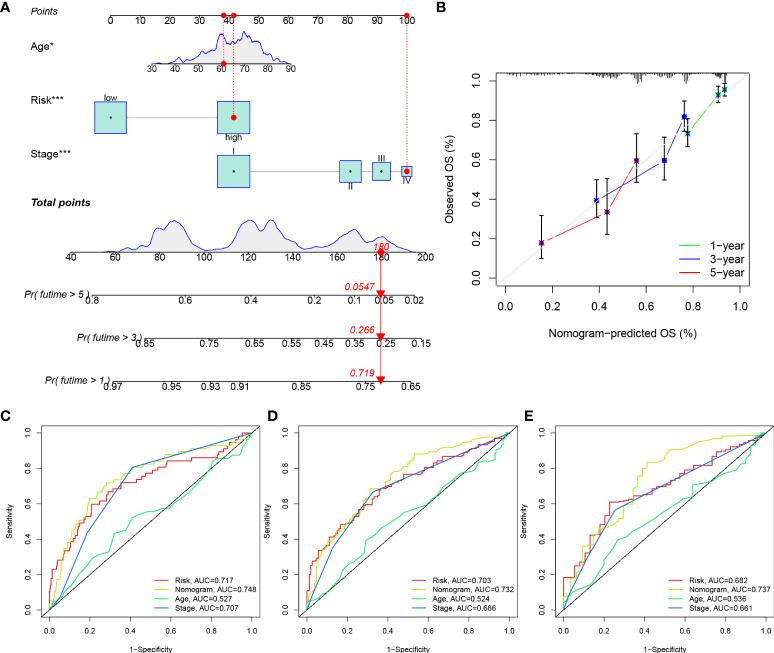
Construction of a nomogram. **(A)** Construction of a nomogram based on risk, age and stage. **(B)** Calibration curves of the nomogram in predicting OS of TCGA-LUAD patients. ROC curves of the nomogram, risk, stage and age in predicting 1 year- **(C)**, 3 years- **(D)** and 5 years- **(E)** OS of TCGA-LUAD patients.

Considering the inconvenience of risk score in predicting OS in patients with LUAD, a nomogram was developed to predict 1-, 3-, and 5-year OS rates by integrating the risk score, age and clinicopathological parameters (**Figure 11A**). The performance of the constructed nomogram in TCGA-LUAD cohort was comparable to an ideal model (**Figure 11B**). We further constructed ROC curves to evaluate the performance of nomogram, risk, stage and age in predicting 1-, 3- and 5-year OS (**Figures 11C–E**). The nomogram always showed the best performance in predicting the 1-, 3- and 5-year OS rates, followed by risk and stage.

### Analysis of Gln metabolism at the level of single cell

**Figure 12:**
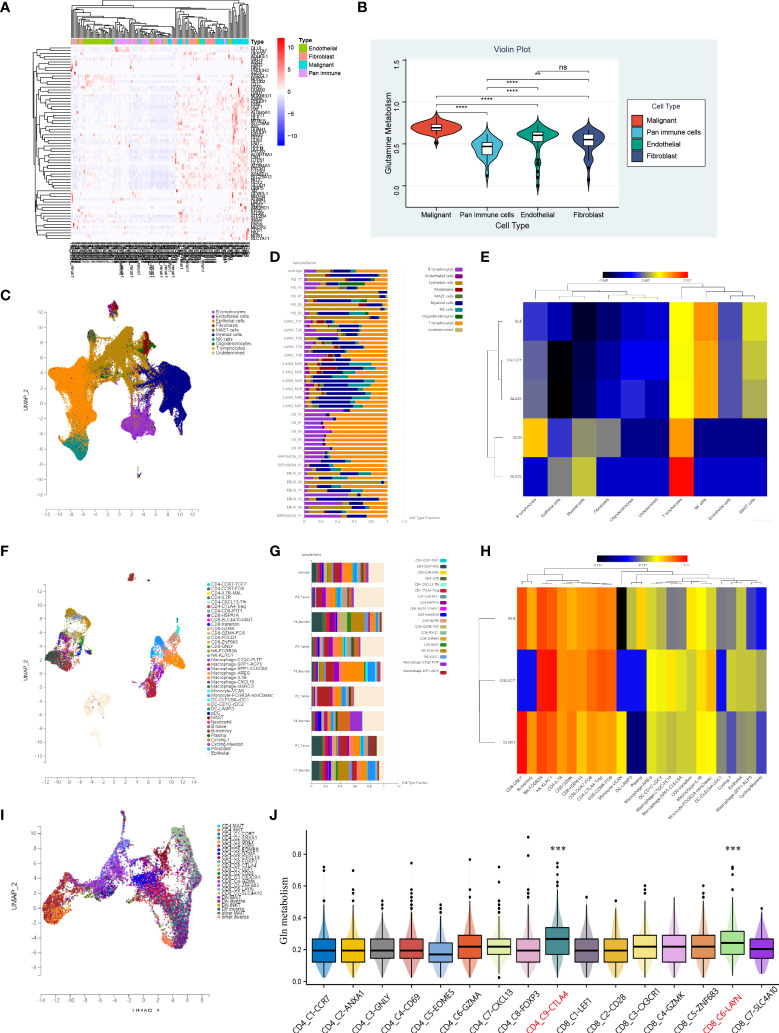
Characteristic of Gln metabolism in TME. **(A)** Expression of identified Gln metabolism regulators in malignant cells, endothelial cells, fibroblasts and pan-immune cells. **(B)** Difference of Gln metabolism levels in malignant cells, endothelial cells, fibroblasts and pan-immune cells. **(C)** The distribution of immune cell clusters in UMAP plot of GSE131907. **(D)** Cell type fraction of each sample in GSE131907. **(E)** Expression of key Gln metabolism regulators in immune cells of GSE131907. **(F)** The distribution of immune cell clusters in UMAP plot of GSE117570. **(G)** Cell type fraction of each sample in GSE117570. **(H)** Expression of key Gln metabolism regulators in immune cells of GSE117570. **(I)** The distribution of T cell clusters in UMAP plot. **(J)** Level of Gln metabolism in 16 distinct T cells. “**” means that p < 0.01; "“***” means that p < 0.001; ****” means that p < 0.0001; no significance.

To investigate the differences in Gln metabolic activity of various cell types in LUAD, we performed an in-depth analysis of public single-cell sequencing data of lung cancer. We developed a heatmap to present the expression of Gln metabolism-related genes in four types of major cells that constitute the TME, including flow-sorted malignant cells, endothelial cells, immune cells and fibroblasts (**Figure 12A**). Gln metabolism-related genes were most significantly upregulated in malignant cells, followed by fibroblasts, while the lowest expression of Gln metabolism was observed in immune cells (**Figure 12A**). The ssGSEA revealed the highest level of Gln metabolism in malignant cells, and the least activity of Gln metabolism in infiltrating immune cells (**Figure 12B**).

To further investigate the differences in Gln metabolism of infiltrating immune cells in the TME, 208506 lung adenocarcinoma cells from 58 specimens were clustered and defined into 10 cell types, including B lymphocytes, endothelial cells, epithelial cells, fibroblasts, mast cells, myeloid cells, NK cells, oligodendrocytes, T lymphocytes, and undetermined cells (**Figure 12C**). Cell type fraction of each sample is presented in **Figure 12D**. A heatmap was plotted to show the expression of key regulators of Gln metabolism (**Figure 12E**). Compared with other cells, T lymphocytes exhibited the most active Gln metabolism. To further validate our findings, 9705 NSCLC cells from GSE117570 were also clustered and defined (**Figure 12F**). Cell composition is presented in **Figure 12G**. Consistently, the key regulators of Gln metabolism were significantly overexpressed in a variety of T cells, revealing a relatively active Gln metabolism in infiltrating T cells (**Figure 12H**). Subsequently, we used single-cell sequencing data of T cells (GSE99254) to investigate the heterogeneity of Gln metabolism in various types of T cells in NSCLC (**Figure 12I**). Based on ssGSEA, exhausted CD8 T cells (C6-LAYN) and suppressive Tregs (C9-CTLA4) were shown to express the most active Gln metabolism compared with other T cells (**Figure 12J**). Interestingly, exhausted CD8 T cells and suppressive Tregs are also key target cells for immune checkpoint inhibitor (ICI) therapy.

### EPHB2 affects the biological behaviors of LUAD cells *in vitro*

We performed differential expression analysis of the 10 genes in pan-cancer risk score (**Supplementary Figure 3**). Among the 10 genes, EPHB2 showed the most significant difference between normal and tumor cells of all cancers and was significantly overexpressed in tumors. However, the biological role of EPHB2 in LUAD was rarely studied. We subsequent performed a series of experiments to elucidate the role of EPHB2 in LUAD.

**Figure 13:**
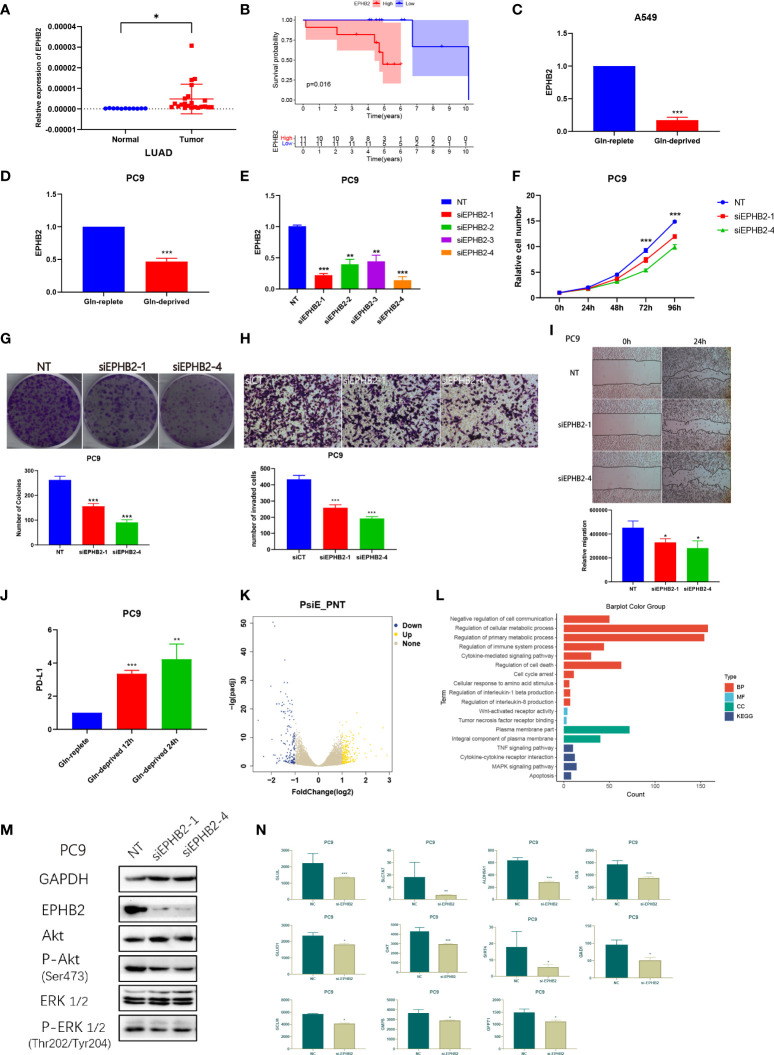
EPHB2 affects the biological behaviors of LUAD cells *in vitro*. **(A)** Expression of EPHB2 in normal and tumor specimens. **(B)** Survival analyses between low and high EPHB2 groups in LUAD cohorts. Expression of EPHB2 with treatment of Gln-replete and Gln-deprived in A549 cell line **(C)** and PC-9 cell line **(D)**. **(E)** QRT-PCR was performed to detect the efficiency of EPHB2-siRNA transfection. **(F)** Growth curves of PC-9 cells treated with EPHB2 knockdown was developed using SRB assay. **(G)** Colony formation assay was conducted to detect the proliferation of PC-9 cells. **(H)** Transwell assay was performed to detect the invasion of PC-9 cells with treatment of EPHB2 knockdown. **(I)** The cell migration of EPHB2 knockdown was detected by wound healing assay in PC-9 cells. **(J)** Expression of PD-L1 with treatment of Gln-replete medium, Gln-deprived medium for 12h and Gln-deprived medium for 24h. **(K)** A volcano map to exhibit differential expressed genes between normal and EPHB2 knockdown treated PC-9 cells. **(L)** GO and KEGG enrichment analysis between normal and EPHB2 knockdown treated PC-9 cells after sequencing. **(M)** GAPDH, EPHB2, AKT, P-AKT (Ser473), ERK1/2, P-ERK1/2 (Thr202/Tyr204) were detected by western blotting in EPHB2 knockdown treated PC-9 cells. **(N)** Expression of key Gln metabolism regulators in normal and si-EPHB2 treated PC-9 cells. "*” means that p < 0.05; “**” means that p < 0.01; "“***” means that p < 0.001.

The expression of EPHB2 in 22 LUAD specimens and 11 normal specimens was detected and EPHB2 was highly expressed in LUAD specimens (**Figure 13A**). Patients with high expression of EPHB2 showed worse overall survivals compared with low EPHB2 group (**Figure 13B**).

To validate the association between EPHB2 and Gln metabolism, we used Gln-deprived/replete medium to culture A549 and PC-9 cells. The expression of EPHB2 was significantly downregulated by Gln-deprived medium in A549 and PC9 (**Figures 13C, D**). We further designed siRNA for EPHB2 knockdown and transfected siRNA into PC9 cells. The siRNA-1 and siRNA-4 were selected for further investigation due to the greater than 70% transfection efficiency (**Figure 13E**). The SRB assay was performed to test the cell proliferation, and the knockdown of EPHB2 significantly inhibited the proliferation of PC9 cells (**Figure 13F**). The number of cell clones was decreased in PC9 cells with EPHB2 knockdown (**Figure 13G**). Transwell assay was performed to investigate the cell invasion: EPHB2 knockdown significantly reduced the invasion of PC9 cells (**Figure 13H**). EPHB2 knockdown also promoted migration of PC9 cells in wound healing assay (**Figure 13I**). In conclusion, knockdown of EPHB2 significantly inhibited cell proliferation, migration and invasion. In addition, surprisingly, the removal of Gln significantly upregulated the PD-L1 expression of PC9 cells, which may indicate the potential therapeutic role of combining Gln metabolism inhibitors with PD-L1 inhibitors (**Figure 13J**).

To explore the regulation of downstream signaling by EPHB2, we knocked down EPHB2 in PC9 cells, followed by transcriptome sequencing, which revealed 565 DEGs, which were screened out with FC > 2 and P< 0.05, including 296 upregulated genes and 269 downregulated genes (**Figure 13K**). GO and KEGG enrichment analysis was performed to identified regulated pathways (**Figure 13L**). EPHB2 was mainly associated with cell communication, cellular metabolic process, regulation of immune, regulation of cell death, cytokine-mediated signaling pathway, response to amino acids, TNF signaling pathway, MAPK pathway and regulation of IL-1β and IL-8 production (**Figure 13L**). Simultaneously, AKT pathway and ERK pathway were verified to be down-regulated when EPHB2 was knocked out, suggesting that EPHB2 is involved in the regulation of these pathways (**Figure 13M**). Besides, 11 key Gln metabolism-related genes were downregulated after treating with EPHB2 knockdown (**Figure 13N**). In particular, the key regulators of Gln metabolism, SLC7A7, GLS, ALDH5A1 and GLUL were significantly downregulated, which indicated significant correlation between EPHB2 and Gln metabolism.

### Effect of EPHB2 on infiltrating immune cells of TME

**Figure 14:**
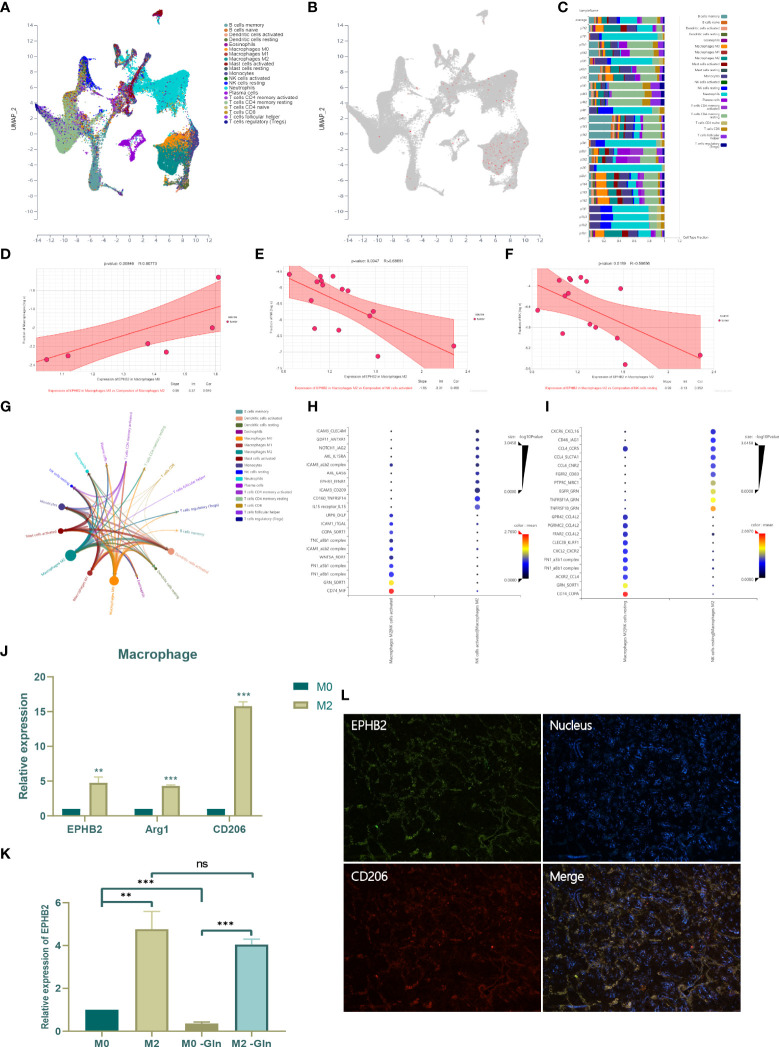
Effect of EPHB2 on infiltrating immune cells of TME. **(A)** The distribution of immune cell clusters in UMAP plot. **(B)** The expression of EPHB2 in distinct clusters of immune cells. **(C)** Cell type fraction of each sample. **(D)** Correlation analysis between expression of EPHB2 in macrophages M0 and composition of infiltrating macrophages M2. Correlation analysis between expression of EPHB2 in macrophages M2 and composition of infiltrating activated NK cells **(E)** and resting NK cells **(F)**. **(G)** Correlation network between tumor infiltrating immune cells. **(H)** The ligand-receptor interaction between macrophages M2 and activated NK cells. **(I)** The ligand-receptor interaction between macrophages M2 and resting NK cells. **(J)** Expression of EPHB2 and macrophages M2 markers in macrophages M0 and M2. **(K)** Expression of EPHB2 in normal macrophages M0, M2 and Gln-deprived macrophages M0, M2. **(L)** Co-localization between EPHB2 and CD206 detected by IF in LUAD specimen. “**” means that p < 0.01; "“***” means that p < 0.001; ns, no significance.

To investigate the expression and role of EPHB2 in immune cells, we selected single cell sequencing data of NSCLCs (GSE127465) for further analysis by clustering and defining 53215 cells into 21 types using algorithm Uniform Manifold Approximation and Projection (UMAP) (**Figure 14A**). EPHB2 was found to be mainly enriched in M0 and M2 macrophages, especially in M2 macrophages, suggesting that EPHB2 may function mainly in macrophages (**Figure 14B**). The cell type fraction of each sample is shown in **Figure 14C**, with M2 constituting almost the highest proportion. We further analyzed the correlation between EPHB2 expression in M0/M2 and the composition of infiltrating immune cells. The expression of EPHB2 in M0 macrophages was significantly and positively correlated with abundance of infiltrating M2 macrophages, which indicated that EPHB2 may be involved in the polarization of M2 macrophages (**Figure 14D**). Besides, the expression of EPHB2 in M2 macrophages was negatively correlated with the abundance of activated NK cells and resting NK cells (**Figures 14E, F**). These results suggest that EPHB2 may be associated with cell communication between M2 macrophages and NK cells. The interaction network of infiltrating immune cells showed that M2 macrophages exhibited the most extensive interactions with other immune cells (**Figure 14G**). The ligand-receptor interaction between M2 macrophages and activated NK cells is presented in **Figure 14H**. Similarly, the ligand-receptor interaction between M2 macrophages and resting NK cells was also investigated (**Figure 14I**). To verify the distribution of EPHB2 in macrophages M0 and M2, we induced THP-1 cells into macrophages M0 and M2, and detected the expression of EPHB2 by qPCR (**Figure 14J**). Compared with M0 macrophages, M2 macrophages showed a significant upregulation of EPHB2, accompanied by significant upregulation of the markers of M2. We further used Gln-deprived medium to culture M0 and M2 macrophages and found that Gln deprivation significantly downregulated EPHB2 expression in M0 macrophages, but did not affect the expression in M2 macrophages (**Figure 14K**). Besides, we also found that EPHB2 was significantly co-expressed with the M2 macrophage marker CD206 in LUAD tissues *via* immunofluorescence (**Figure 14L**). These results suggest that EPHB2 also plays a huge role in macrophages.

## Discussion

Although targeting cancer metabolism to enhance immunotherapy responsiveness is highly promising, the heterogeneity and crosstalk of metabolic pathways between cancer cells and immune cells in TME lead to disruption of normal metabolic pathways in immune cells by strategies to inhibit/alter cancer metabolism (27). Therefore, it is critical to target the appropriate metabolic pathways and molecules to kill tumors without interfering with or even promoting anti-tumor immunity. However, recent studies have shown that JHU083, a broad-spectrum inhibitor of Gln metabolism, effectively kills tumor cells while activating the anti-tumor effects of CD8+ T cells, thereby significantly enhancing the efficacy of anti-PD-1 immunotherapy (15). Meanwhile, another study reported that targeting Gln metabolism increased antitumor immunity in mouse models by upregulating mitochondrial metabolism of CTLs in NSCLC (28, 29). These studies make Gln metabolism an ideal target for improving tumor immunotherapy, but related multi-omics systematic studies are still extremely rare in LUAD and even in other tumors.

Herein, we first defined four patterns based on prognosis-related regulators of Gln metabolism. The four clusters exhibited significantly different prognostic features, Gln metabolism and TME. The immune phenotype gradually changes from “cold” to “hot” sequentially, from clusters C1 to C4, accompanied by an upregulation of the abundance of infiltrating immune cells and activation of the anti-tumor immune pathway. Notably, the “hot” immune phenotype in different clusters is often associated with a survival advantage and low levels of Gln metabolism. Gln is a common metabolic substrate in tumor and immune cells (9), and therefore tumor cells can reduce the anti-tumor effect of Gln-dependent immune cells, such as T cells and DCs, by competing for and depleting Gln. Gln metabolism was shown to mediate the activation of DCs, and coincidentally, low levels of Gln metabolism and highly enriched DCs were present concurrently in cluster C4, followed by upregulation of APC co-stimulation and HLA. These suggest activation of the antigen presenting pathway, which may contribute to the significant upregulation of TIL and T cell co-stimulation in cluster C4. Based on DEGs, patients with LUAD were further classified into three geneClusters. Similar to the previous clusters, the immune phenotype also showed a transition from “cold” to “hot” from geneClusters A to C, and exhibited a similar TME landscape. In addition to DCs, various helper T cells exhibited significant differences, including Th1 and Th2 cells. Studies have shown that Gln deficiency alters Th1 differentiation and converts CD4+ T cells to a Treg phenotype (30). In addition, genetic deletion of the Gln transporter protein ASCT2 impaired Th1 production and function (31). In the group with low Gln metabolism, CD4+ T cells may acquire additional Gln and thus promote Th1 cell differentiation and activation. Th1 mediates anti-tumor immunity mainly by expressing CD40L and secreting cytokines such as INFγ and IL-2 to recruit and activate macrophages and cytotoxic T cells, which may be involved in the upregulation of TIL, macrophages and type II IFN response in geneCluster C (32). In addition, we found that low Gln metabolism in tumors may drive the Th1/Th2 balance toward Th1, which favored anti-tumor immunity (33).

Based on prognosis-related DEGs, we developed a risk score and divided patients with LUAD into low- and high-risk groups. Similarly, the low-risk group was defined as “hot” immunophenotype, corresponding to a survival advantage and lower levels of Gln metabolism, while the high-risk group showed the opposite effect. In the low-risk group, the low levels of tumor Gln metabolism may imply a weaker competitive depletion of Gln, thus allowing immune cells to acquire further Gln and activate anti-tumor effects, which may explain the upregulation of anti-tumor immune cells or pathways such as DCs, TIL, Th1 cells, NK cells, APC co-stimulation, T-cell co-stimulation and type II IFN response. “Hot” immune phenotype was shown to benefit strongly from immunotherapy, which was also validated by the levels of immune checkpoints, TIDE, IPS and immunotherapy cohorts. Patients in low-risk group benefited significantly from immunotherapy, especially following ACT therapy of melanoma cohort and anti-PD-1 antibody treatment of NSCLC cohort. Deletion of glutaminase enhanced the effector differentiation of CAR-T cells (34). Alternatively, no further studies are available to demonstrate that Gln metabolic blockade improves the efficacy of ACT therapy. Although extensive blockade of Gln metabolism has been shown to significantly enhance the efficacy of anti-PD-1 therapy, corresponding studies in LUAD are still lacking. Therefore, the constructed risk model not only facilitates the differentiation of the efficacy of immunotherapy, but also provides an important reference for Gln blockade combined with immunotherapy. In addition, the risk model was used to significantly differentiate patient prognosis in 23 different cancers, indicating the generalizability of the model.

Gln metabolism was shown to be involved in multiple cancer progression as shown in our study. Gln metabolism was significantly and positively correlated with TNM and stage (**Figures 7G–J**). We performed single-cell sequencing analysis to describe the landscape of Gln metabolism in TME. Consistent with previous results, tumor cells exhibited significantly activated Gln metabolism compared with immune cells or fibroblasts. However, in two independent single-cell sequencing analyses of LUAD, T cells exhibited relatively higher active Gln metabolism compared with other immune cells. Although Gln metabolism has been reported to be involved in T cell differentiation and activation, the landscape of Gln metabolism in tumor-infiltrating T cells remains elusive (30). Therefore, we further extracted and analyzed single-cell sequencing data targeting lung cancer-infiltrating T cells. Surprisingly, exhausted CD8 T cells and suppressive Tregs exhibited the most active Gln metabolism compared with other 14 types of T cells, and represent key target cells in anti-PD1 and anti-CTLA4 immunotherapy, respectively (35, 36). These results suggest the feasibility of utilizing Gln metabolism inhibitors combined with immunotherapy. Indeed, due to the robust plasticity of T cell metabolism, the blockade of Gln metabolism increases T cell proliferative capacity and anticancer activity, in addition to preventing exhaustion *via* T cell metabolic reprogramming (15).

To further characterize the genes used in the model, we performed differential pan-cancer analysis, showing that EPHB2 is most differentially and highly expressed in the vast majority of tumors (**Supplementary Figure 3**). EphB2 is a significant member of the Eph receptor family, which has been verified to regulate the malignant progression of various tumors through different signaling pathways. In hepatocellular carcinoma, EPHB2 enhances cancer stem cell properties and drive sorafenib resistance by activating SRC/AKT/GSK3β/β-catenin signaling cascade. Moreover, EPHB2 mediated malignant progression of medulloblastoma by regulating ERK, P38 and mTOR pathway (37, 38). Although studies have shown that EPHB2 is involved in the malignant progression of various cancers, its role in LUAD has yet to be investigated (37). In the present study, we found that EPHB2 was closely associated with malignant progression of LUAD, promoting proliferation, invasion and migration of LUAD cells. Simultaneously, EPHB2 has been verified to be involved in the regulation of AKT pathway and ERK pathway, which may be the potential mechanism for promoting the malignant progression of LUAD by EPHB2. Interestingly, Gln deprivation significantly downregulated EPHB2 expression, and knockdown of EPHB2 in turn downregulated key regulators of Gln metabolism, such as GLS, GLUL, SLC7A7 and GLUD1. Meanwhile, the results of enrichment analysis after transcriptome sequencing showed that EPHB2 was associated with cellular metabolic regulation and response to amino acid stimulus. Therefore, we speculate that EPHB2 may be involved in the Gln metabolic pathway, which has yet to be reported.

Based on transcriptome sequencing analysis, EPHB2 was also significantly associated with cell communication and immune regulation. Although previous studies reported that EPHB2 promoted monocyte activation and T-cell migration, studies investigating the regulation of tumor immunity by EPHB2 are still unavailable (39, 40). In our study, we found that EPHB2 was mainly enriched in macrophages, especially in M2 types. EPHB2 expression in M0 macrophages enhanced the levels of M2 macrophages, and the expression of EPHB2 in M2 macrophages reduced the composition of activated and resting NK cells (**Figure 14**). These results suggest that EPHB2 may promote M2-like polarization and also mediate the interactions between M2 macrophages and NK cells, which in turn suppress NK cell infiltration or proliferation. Previous studies revealed that the expression of EPHB2 was significantly correlated with trans-differentiation of monocytes into macrophages by upregulating CCL2 and IL-8 (40). However, no previous study explored the function of EPHB2 in M2 macrophages, which was precisely the focus of our study. Previous research revealed that Gln metabolism positively regulated M2-like polarization of macrophage, which may be the potential mechanism of regulating M2-like polarization by EPHB2 (13).

However, our study did not elucidate the mechanism of EPHB2 in LUAD cells and M2 macrophages, which will be addressed in future studies.

In conclusion, based on the regulators of Gln metabolism, we finally constructed a Gln metabolism-related risk model to accurately predict the prognosis of patients with LUAD and even multiple cancers as well as the efficacy of multiple immunotherapies. In addition, we described the Gln metabolism of cells in TME at the single-cell level. Finally, EPHB2, a Gln metabolism-related molecule in the model was shown to promote the malignant progression of LUAD cells and also play an essential role in M2 macrophages.

## Data availability statement

The data presented in the study are deposited in the GEO repository, accession number GSE209973.

## Ethics statement

Our research obtained approval from Biomedical Research Ethic Committee of Shandong Provincial Hospital (SWYX: NO.2022-262).

## Author contributions

JD contributed to the designing and supervising the study, and correspondence. Co-authors JL and HS analyzed the data and completed the manuscript. WG, HZ, YW, and GM helped to search for references and offered guidelines of statistical methods. All authors have read and approved the final version to be published.

## Funding

This work is supported by Natural Science Foundation of Shandong Province (Grant No. ZR2020QH215) and Clinical Medicine Science and Technology Innovation Plan of Jinan (Grant No. 202134042).

## Acknowledgments

We expressed our sincere thanks to the research groups of Prof. Xin Gao (State Key Laboratory of Experimental Hematology, National Clinical Research Center for Blood Diseases, Institute of Hematology and Blood Diseases Hospital, Chinese Academy of Medical Sciences and Peking Union Medical College, Tianjin, China) for providing help with single-cell sequencing analysis.

## Conflict of interest

The authors declare that the research was conducted in the absence of any commercial or financial relationships that could be construed as a potential conflict of interest.

## Publisher’s note

All claims expressed in this article are solely those of the authors and do not necessarily represent those of their affiliated organizations, or those of the publisher, the editors and the reviewers. Any product that may be evaluated in this article, or claim that may be made by its manufacturer, is not guaranteed or endorsed by the publisher.

## Abbreviations

Gln, glutamine; LUAD, Lung Adenocarcinoma; TME, Tumor microenvironment; TCGA, The Cancer Genome Atlas; DEGs, Differentially expressed genes; GEO, Gene-Expression Omnibus; NK cells, Natural killing cells; NSCLC, Non-small Cell Lung Cancer; OS, overall survival; TPM, transcripts per kilobase million; FPKM, Fragments Per Kilobase of exon model per Million mapped fragments; MSigDB, Molecular Signatures Database; ROC, receiver operating characteristic; GSVA, Gene Set Variation Analysis; ssGSEA, Single sample gene set enrichment analysis; MAF, mutation annotation format; TMB, tumor mutation burden; IPS, Immunophenoscore; ACT, adoptive T cell therapy; TIDE, Tumor immune dysfunction and exclusion; INFG, interferon gamma; MDSCs, Myeloid-derived suppressor cells; MSI, Microsatellite instability; CAF, cancer associated fibroblast; TAM, Tumor-Associated Macrophages; GEPIA, Gene Expression Profiling Interactive Analysis; CNV, copy number variations; DCs, Dendritic cells; TILs, Tumor infiltrated lymphocytes; APC, antigen-presenting cell; HLA, human leukocyte antigen; PCA, Principal Components Analysis; AIC, Akaike information criterion; ICI, Immune checkpoint inhibitor; UMAP, Uniform Manifold Approximation and Projection; GO, Gene ontology; KEGG, Kyoto Encyclopedia of Genes and Genomes.
