# Supplementary material for: Prediction of prognosis, immunogenicity and efficacy of immunotherapy based on glutamine metabolism in lung adenocarcinoma
Source: Front Immunol. 2022 Aug 11;13:960738. doi: 10.3389/fimmu.2022.960738 (PMC9403193; doi:10.3389/fimmu.2022.960738)
Supplement: Supplementary file 1 [file DataSheet_1.docx]

Supplementary Material


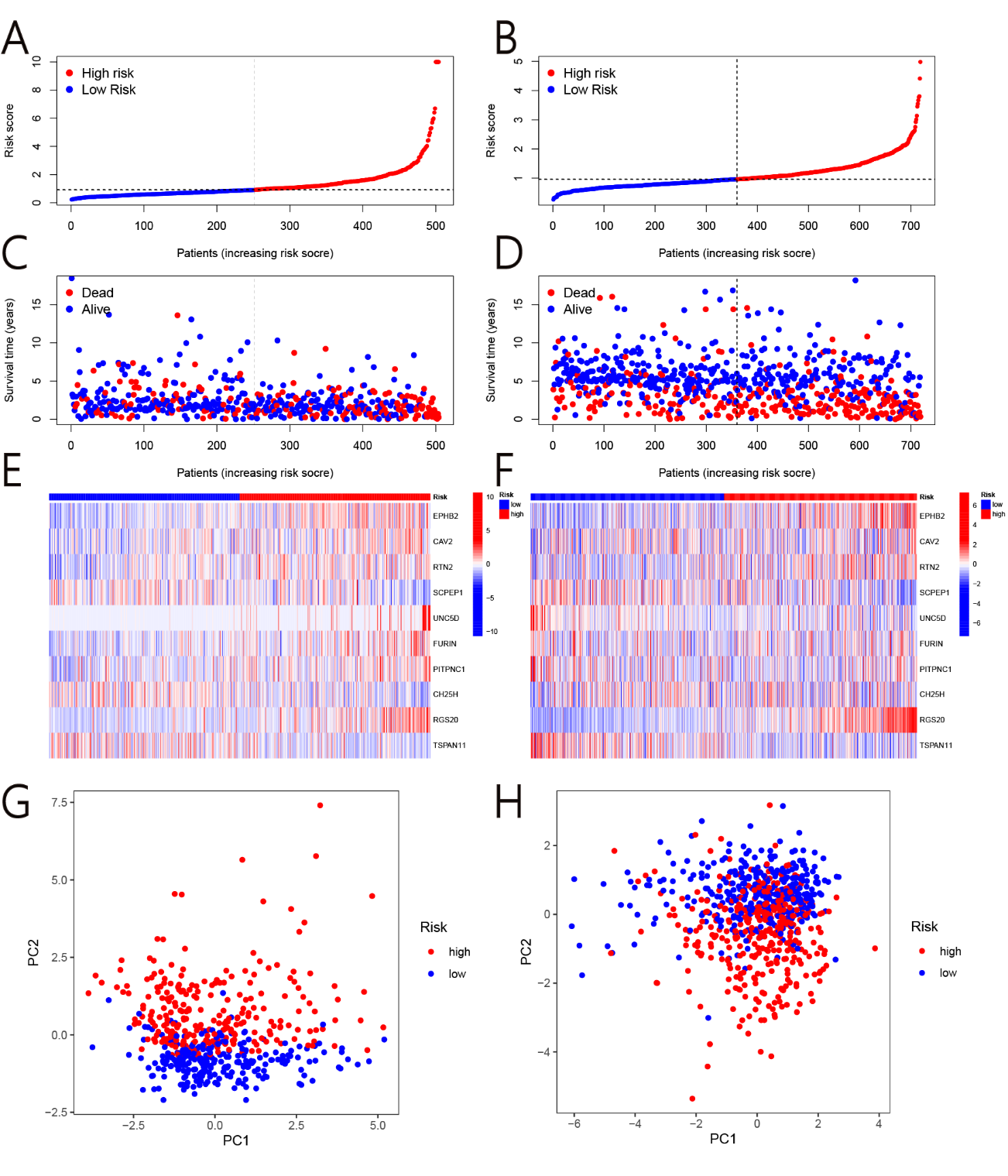


**Figure S1. Distribution of risk score, survival status and gene expression.**

Distribution of risk score in training cohort (A) and testing cohort (B). Distribution of survival status in training cohort (C) and testing cohort (D). Expression of ten genes between high- and low-risk groups in training cohort (E) and testing cohort (F). PCA for the transcriptome profiles of two risk groups in training cohort (G) and testing cohort (H).


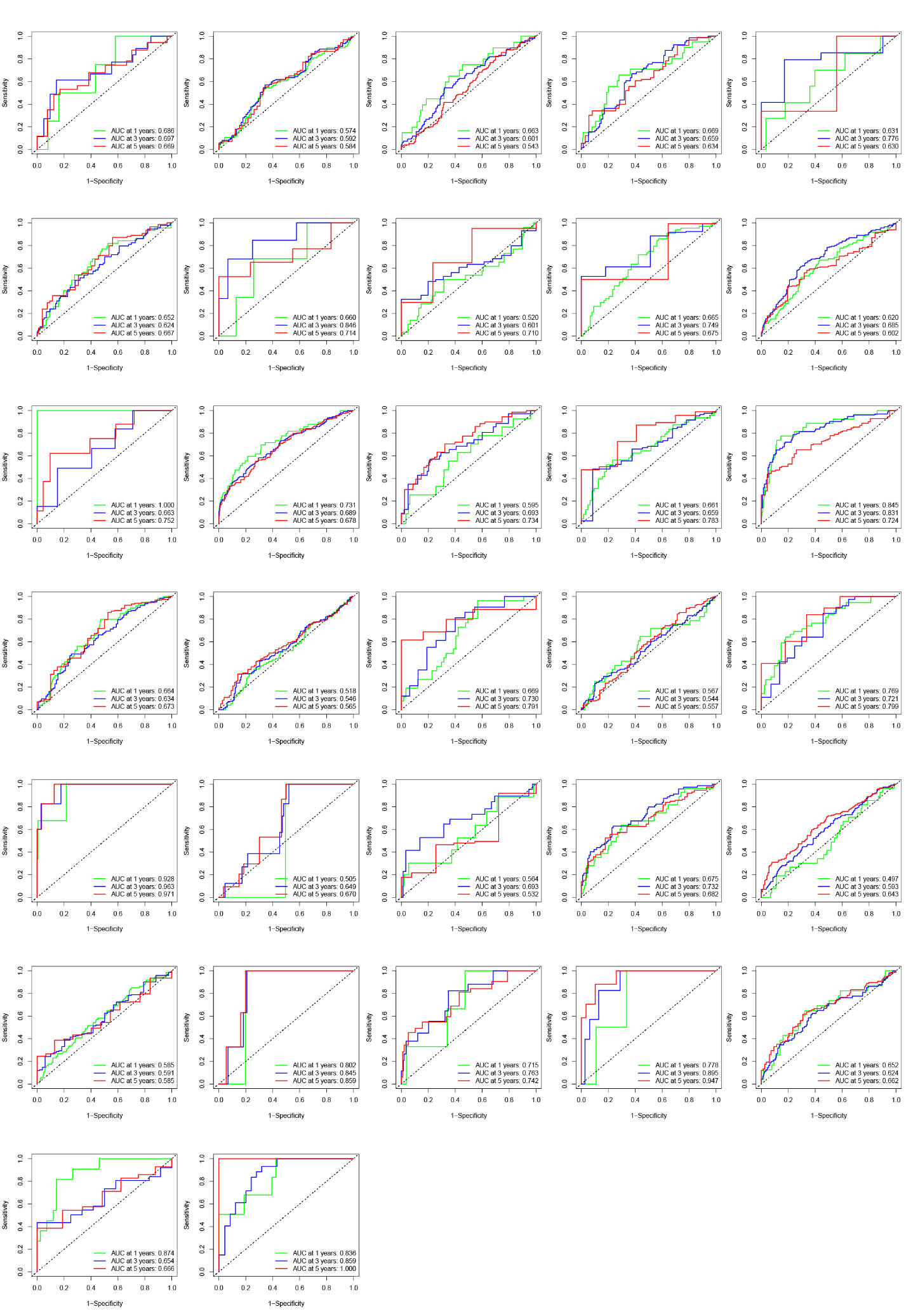


**Figure S2. ROC curves of risk score in predicting prognosis of pan-cancer.**


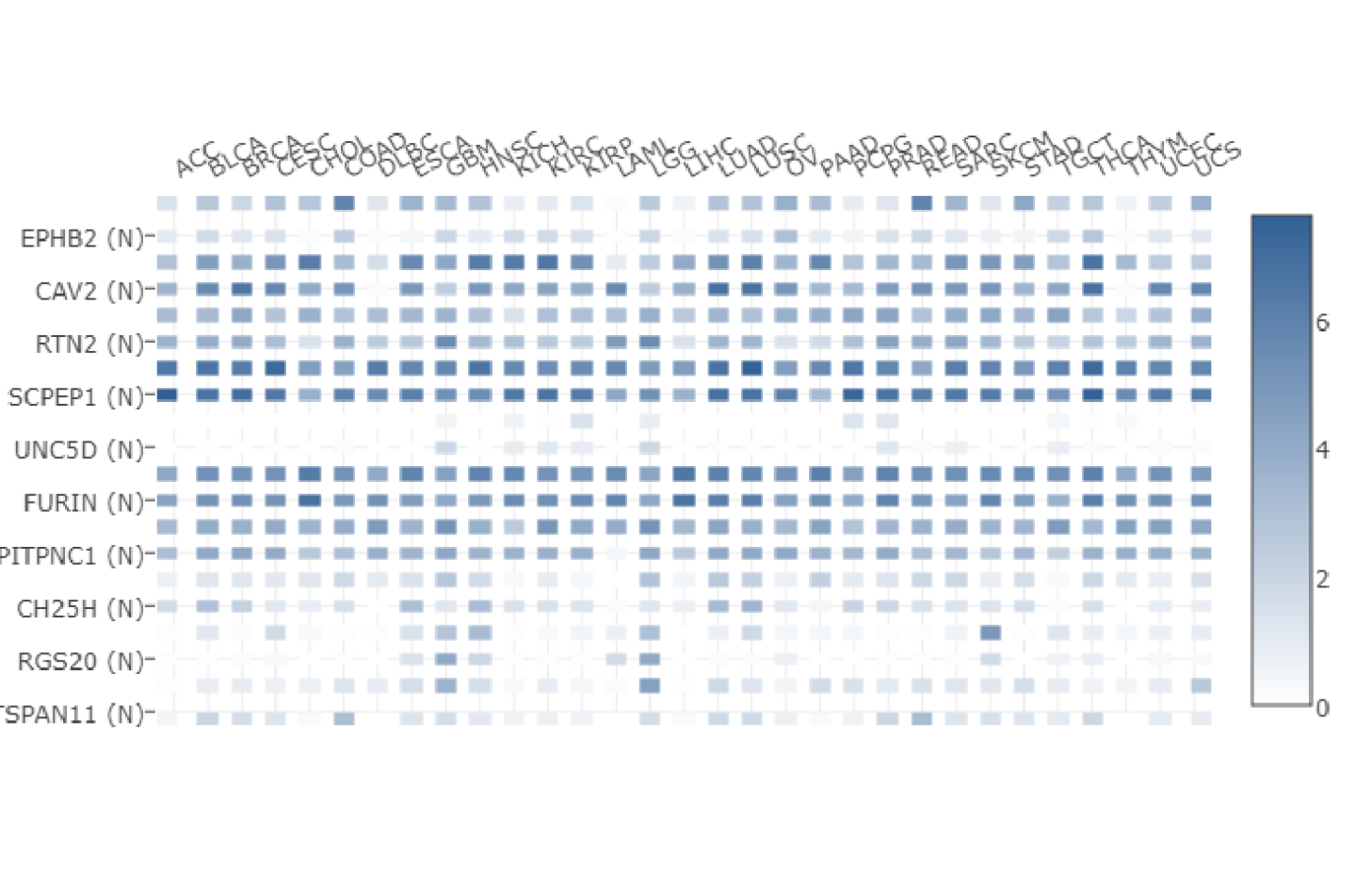


**Figure S3. Gene expression of risk model in pan-cancer.**
