# Supplementary material for: Prediction of prognosis, immunogenicity and efficacy of immunotherapy based on glutamine metabolism in lung adenocarcinoma
Source: Front Immunol. 2022 Aug 11;13:960738. doi: 10.3389/fimmu.2022.960738 (PMC9403193; doi:10.3389/fimmu.2022.960738)
Supplement: Supplementary file 2 [file Table_1.docx]

**Table S1.** **Primers used in this study**

| **gene** | **primer-F (5'-3')** | **primer-R (5'-3')** |
| --- | --- | --- |
| 18S | CAGCCACCCGAGATTGAGCA | TAGTAGCGACGGGCGGTGTG |
| EPHB2 | TGTCGGACTTTGGGCTCTCAC | AACTTCCGGTACTGGATGGCTTC |
| CD274 | GCTATGGTGGTGCCGACTACAA | GGTGGTGGTCTTACCACTCAGGA |
| CD206 | GCCCGGAGTCAGATCACACA | AGTGGCTCAACCCGATATGACAG |
| ARG1 | CTGGCAAGGTGGCAGAAGTC | ATGGCCAGAGATGCTTCCAA |
|  |  |  |
